# Key regulatory genes in sugar beet's defense against curly top virus identified by network analysis and qRT-PCR

**DOI:** 10.1016/j.bbrep.2025.102214

**Published:** 2025-08-19

**Authors:** Zeinab Porameri, Abozar Ghorbani, Zahra Mirsoleymani, Marzieh Karimi, Mahsa Rostami, Seyed Ali Hemmati

**Affiliations:** a Department of Plant Protection, Faculty of Agriculture, Shahid Chamran University of Ahvaz, Ahvaz, Iran; b Nuclear Agriculture Research School, Nuclear Science and Technology Research Institute (NSTRI), Karaj, Iran; c Institute of Biotechnology, Shiraz University, Shiraz, Iran

**Keywords:** BCTV, Gene network, BCTIV, Sugar beet, Hub genes

## Abstract

Curly top disease, caused by Beet Curly Top Virus (BCTV), is a major threat to sugar beet (*Beta vulgaris*), resulting in significant yield losses. This study integrates RNA sequencing, gene network analysis, and experimental validation to uncover key regulatory genes involved in plant responses to viral infection. Network analysis identified nine central hub genes associated with fatty acid metabolism, stress adaptation, and transcriptional regulation. Meanwhile, functional enrichment analysis highlighted chloroplast-associated immune signaling, oxidative stress modulation, and secondary metabolite biosynthesis as critical defense mechanisms. Due to the genomic similarities between BCTV and Beet Curly Top Iran Virus (BCTIV), BCTIV was selected to investigate whether conserved molecular responses exist in sugar beet infected by these phylogenetically related viruses. The upregulation of hub genes - Su1 (EMB3147), Su2 (FRS5), and Su3 (LACS9)- under BCTIV infection was found to mirror patterns observed in BCTV-infected plants, suggesting convergent defense mechanisms against both viruses. A strong correlation (R^2^ = 0.995) between qRT-PCR and RNA-Seq data further confirmed that the close genomic proximity of BCTIV to BCTV results in analogous transcriptional reprogramming in the host, supporting the broader relevance of these findings for curly top disease management.

## Highlights

• Hub genes linked to virus defense identified.
• Chloroplasts and stress pathways involved.
• BCTIV mimics BCTV response in sugar beet.
• qRT-PCR confirms RNA-Seq findings.
• Hormonal signaling drives defense genes.

## 1 Introduction

Curly top disease, caused by Beet Curly Top Virus (BCTV) and Beet Curly Top Iran Virus (BCTIV), represents a major challenge to sugar beet (*Beta vulgaris*) and other dicotyledonous crops, leading to severe yield losses. These viruses, members of the *Geminiviridae* family, are transmitted by leafhoppers and seeds, enabling rapid disease to spread across agricultural regions [1]. BCTV and BCTIV show remarkable functional convergence, particularly in their ability to manipulate host cell cycle regulators. Notably, co-infection with BCTV and BCTIV intensifies disease symptoms [2]. Moreover, by hijacking the host's cellular machinery, BCTV induces profound metabolic and regulatory disruptions, interfering with key pathways in primary metabolism, immune signaling, and stress adaptation. This highlights the complexity of viral interactions and their impact on host-pathogen dynamics [3].

Viruses extensively reprogram host metabolism to support their replication, disrupting key pathways such as fatty acid biosynthesis, carbohydrate metabolism, and redox homeostasis [4]. These changes have been well-documented in other geminivirus infections, including Tomato yellow leaf curl virus (TYLCV), where disruptions in salicylic and jasmonic acid biosynthesis and the signal transduction of phytohormones have been observed [5]. In sugar beets, BCTV infection triggers a complex array of host defense responses. These responses are linked to mechanisms such as danger peptide signaling, as seen in BSCTV infection, where PEPR2 recognition of C4 suppresses viral spread. Such findings highlight the intricate interplay between geminivirus symptoms and plant immune pathways [6,7]. These observations suggest that understanding metabolic reprogramming is critical for deciphering plant-virus interactions and developing effective resistance strategies [4].

Advancements in bioinformatics and omics technologies have revolutionized the study of plant-virus interactions, with RNA sequencing (RNA-Seq) and gene network analysis emerging as powerful tools for identifying key regulatory genes. Hub genes, central regulators in biological networks, play a crucial role in orchestrating plant stress responses. These hub genes can be effectively identified through RNA-Seq and gene network analysis, bridging molecular insights with functional responses. Studies in *Arabidopsis thaliana* and *Nicotiana benthamiana* have identified key hub genes in viral resistance [8,9]. The functional role of key genes in sugar beet's defense against curly top viruses remains largely unexplored. This gap underscores the need for systematic investigations into the molecular mechanisms underlying sugar beet's response to BCTV and BCTIV infections. Here, we integrate RNA-Seq, protein-protein interaction (PPI) analysis, and quantitative real-time polymerase chain reaction (qRT-PCR) validation to identify critical hub genes involved in sugar beet's response to BCTV and BCTIV infections. By examining differentially expressed genes (DEGs) and their associated biological pathways, we aim to uncover the molecular mechanisms governing plant resistance, with a focus on lipid metabolism, oxidative stress response, and transcriptional regulation. These insights will contribute to the development of disease-resistant sugar beet cultivars and inform novel strategies for managing curly top disease.

## 2 Materials and methods

### 2.1 Data collection of the genes involved in BCTV infection

Genes involved in sugar beet complex virus infection were identified by analyzing gene expression profiles from previous studies [10]. Genes demonstrating more than two-fold upregulation or downregulation were selected for gene network analysis.

### 2.2 PPI networks and hub analysis

To investigate the interactions among the selected genes, a protein-protein interaction (PPI) network was created using STRING v10 (http://string-db.org) with a minimum interaction confidence score of 0.150 (low confidence). The resulting PPI dataset was then imported into Cytoscape v3.9.1 for topological refinement and modular analysis. Key genes in the network were identified using CytoHubba (v0.1), a Cytoscape plugin designed to rank critical nodes in biological networks.

Four different computational algorithms implemented in the CytoHubba plugin were applied to identify hub genes: (1) Bottleneck, which prioritizes nodes with high betweenness centrality essential for inter-module communication; (2) EPC (Edge Percolated Component), which assesses node indispensability by analyzing network fragmentation upon node removal; (3) Radiality, which evaluates a node's centrality based on its distance to all other nodes in the network; and (4) Clustering Coefficient, which ranks nodes by their local clustering coefficient—a measure of how densely connected a node's neighbors are. Nodes with high clustering coefficients are likely part of tightly knit regions of the network, often reflecting functionally related gene groups. These algorithms were run independently, and the top-ranked genes from each method were integrated to identify consensus hub genes. The resulting hub genes and their interactions were then visualized as a subnetwork using Cytoscape for better biological interpretation. The identified hub genes and their interactions within the network were visually represented as a subnetwork for clearer interpretation.

### 2.3 Gene ontology and enrichment analysis

Pathway analysis using the Kyoto Encyclopedia of Genes and Genomes (KEGG) was performed on subnetwork genes, and the STRING web-based database was utilized for gene ontology (GO) analysis, including molecular function (MF), cellular components (CC), and biological process (BP).

### 2.4 Promoter analysis of hub genes

The 1 kbp upstream regions of the hub genes were obtained from Ensembl Plants Web Services (http://plants.ensembl.org). Conserved motifs within these sequences were identified using MEME Suite (version 5.4.1) (meme.nbcr.net/meme/intro.html), with default settings applied except for P-value and E-value thresholds, which were set at <0.01. To filter out redundant motifs and recognize known *cis*-regulatory elements (CREs), the Tomtom tool (version 5.4.1) (http://meme-suite.org/tools/tomtom) was utilized, leveraging the JASPAR CORE 2022 database under P-value and E-value thresholds of <0.01 and < 0.1, respectively. Additionally, the GoMo tool (http://meme-suite.org/tools/gomo) was employed to predict the potential functional roles of the identified motifs [11].

### 2.5 Validation of key gene expression through qRT-PCR analysis

To validate the expression of key genes, plants of the Brigitta cultivar were grown in a growth chamber at 26–28 °C with a 16-h light/8-h dark cycle using a cocopeat-perlite mixture. After two weeks of growth, when plants reached the four-leaf stage, healthy plants were agro-inoculated with *Agrobacterium tumefaciens* strain C_58_ carrying BCTV, BCTIV, and BCTV-BCTIV clones. The viral clones used in this study were obtained from the Shahid Chamran University of Ahvaz, Iran. Leaf samples were collected for analysis one week after inoculation [12]. The experimental design was completely randomized, with three infected pots per treatment and three virus-free control pots per biological replicate. Genomic DNA was extracted from leaf tissue using the CTAB method [13], and the presence of viral DNA was confirmed by PCR using specific primers [14]. Subsequently, total RNA was extracted from infected leaves using the Column RNA Isolation Kit (Denazist Azma Company, Iran) according to the manufacturer's protocol, and cDNA synthesis was performed using the Thermo Scientific Reverse Transcriptase First-Strand cDNA Synthesis Kit (Smobio, Taiwan). The qRT-PCR primers were designed using Primer3 tools (https://primer3.ut.ee/) and validated for specificity and efficiency using PrimerBiosoft (https://www.premierbiosoft.com/). Primers were designed for all hub genes from the gene network analysis in *B. vulgaris* in response to the viral infection. The qRT-PCR reactions were performed using the CyberGreen kit (Ampliqon, Denmark) on a Bioneer Real-Time Quantitative PCR instrument (South Korea). Each reaction had a final volume of 12.5 μL, consisting of 0.5 μL forward and reverse primers (10 μM), 6.25 μL SYBR Green (2X), 2 μL cDNA, and 3.25 μL sterile water. Thermal cycling conditions included an initial denaturation at 95 °C for 2 min, followed by 40 cycles of denaturation at 95 °C for 20 s, annealing at 60 °C for 20 s, and extension at 72 °C for 20 s. A final extension was performed at 72 °C for 5 min, followed by melting curve analysis to confirm specificity. The list of primers used for real-time PCR is shown in Table 1.

**Table 1:** Primers used for qRT-PCR to validate RNA-Seq results.

Initiator name	Primer sequence	Length of replication fragment (bp)
su1 (EL10Ac7g15913)	F: AACAGAGATCAGGACACCGC	110
	R: CTGGAGAAGTCACCTGTCGT	
su2 (EL10Ac3g06581)	F: GCTGGTGGTGCTGAGAGTAC	122
	R: GGCATCCACTCCCTTGTTCC	
Su3 (EL10Ac5g12641)	F: TAGAAAGGTCCGAGCAGTGC	137
	R: TCTCGGTGAGCCCATAACCT	
EL10Ac4g09232	F: CAGTGGGTGAGGGAGAGAGA	179
	R: CCTCTTCCGTCATGCTGAGC	
EL10Ac7g17778	F: ACAGCAAGATTGTCAGCCGG	132
	R: GCGCCTTCCAAGACTTCTCC	
EL10Ac7g17862	F: GTGACCGGCAATCTGTGGAC	100
	R: CCATCTTCCATCTCCAGCGC	
EL10Ac6g14637	F: ATGCTGTCAGTGGTGGCGTA	112
	R: CCTGTCCACCGCAAACCTAC	
EL10Ac5g12946	F: AGGTGGGTTTGAGCTGGGTA	130
	R: CAGCCGCATCCATATCCACC	
Actin	F: AATGTTCCCTGGTATTGCTGAC	304
	R: CACTTTCTGTGGACGATTGATG	

The relative gene expression levels of the target genes were calculated using the 2^−ΔΔCT^ method with the actin gene as the internal reference (Livak & Schmittgen, 2001; Pfaffl et al., 2002). Data analysis was performed in Excel, and results were expressed as fold changes relative to the control group for three selected genes (su1, su2, su3) as study in both viruses. To assess the correlation between RNA-Seq and qRT-PCR results, the expression levels of the hub genes were analyzed using Python 3. A custom script was implemented for statistical analysis, and the correlation results were visualized using the matplotlib library.

## 3 Results and discussion

### 3.1 PPI networks and hub genes analysis

**Fig. 1:**
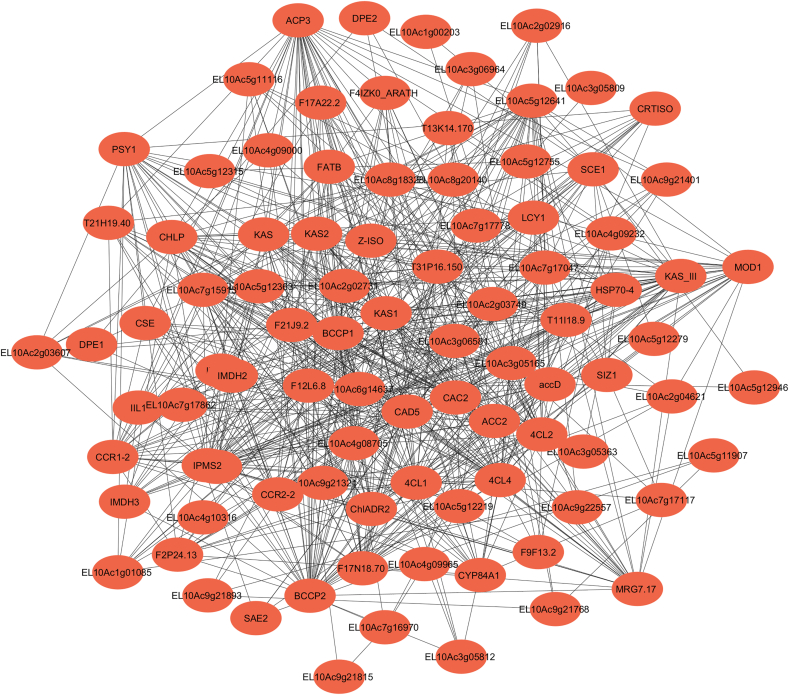
Gene network analysis of differential expression genes in response to Beet curly top virus in sugar beet using String database (https://string-db.org/Version: 12.0) Number of nodes: 99/number of edges: 637/average node degree: 12.9- avg. Local clustering coefficient: 0.524/expected number of edges: 350 -PPI enrichment p-value: <1.0e-16.

This study analyzed RNA-Seq data from sugar beet infected with BCTV to reconstruct PPI networks and identify key hub genes involved in plant responses to viral infection. Using STRING (v12.0) with a low-confidence interaction threshold (score ≥0.15), the resulting PPI network consisted of 99 nodes and 637 edges, showcasing high connectivity and modularity, with an average node degree of 12.9 and a clustering coefficient of 0.524 (PPI enrichment p < 1.0e-16), as visualized in Fig. 1. This broader range of potential interactions was selected due to the limited annotation of PPIs in non-model plants like sugar beet.

**Fig. 2:**
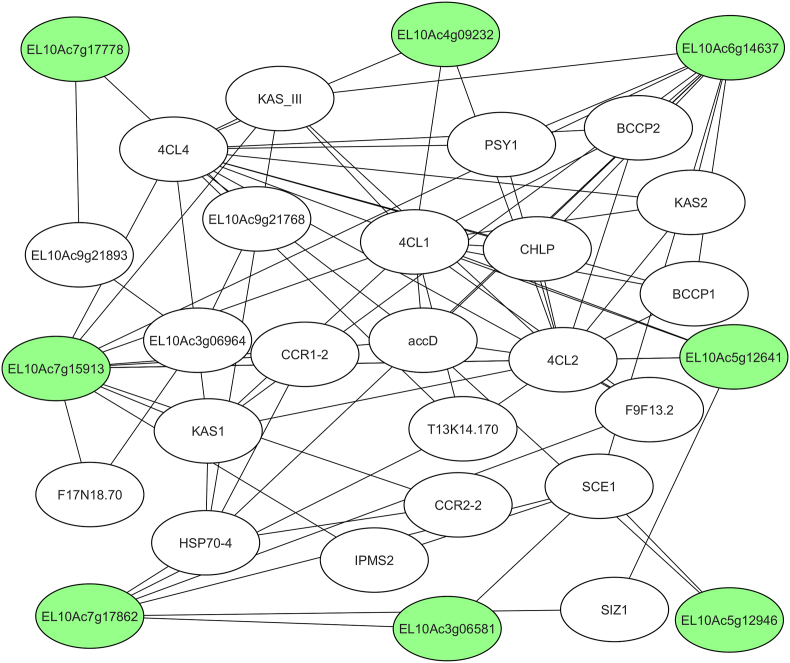
Subnetwork genes in *Beta vulgaris* in response to Beet Curly Top Virus infection, visualized using the CytoHubba App. Green nodes represent hub genes, while white nodes correspond to known interacting partners.

To identify key regulatory genes within this network, several CytoHubba algorithms were applied, resulting in the identification of several hub genes critical to the response to viruses in sugar beet. The genes were ranked based on their interaction significance and functional relevance, as shown in Fig. 2. To robustly identify key regulatory nodes, we employed four distinct CytoHubba algorithms: Bottleneck, EPC, Radiality, and Clustering Coefficient. Each algorithm captures different topological aspects of the network. Specifically, the Clustering Coefficient method highlights nodes whose immediate neighbors are highly interconnected, forming dense local clusters. Such nodes are likely to be involved in similar or cooperative biological functions. By integrating the top candidates across these algorithms, we identified a set of consensus hub genes that play central roles in the sugar beet's response to viral infection. The identified genes were further visualized as a subnetwork to emphasize their functional interactions.

Among the top-ranked hub genes, Expansin-like A3 (EL10AC5g12946), ranked first by the clustering coefficient method, is pivotal in modifying host cellular architecture. Expansins are known to facilitate cell wall loosening, a process that viral pathogens exploit to enhance infectivity [15]. The involvement of this gene underscores the importance of structural changes in the plant's response to viral infection. Additionally, the TFIIE alpha subunit (EL10AC4g09232), which also ranked first in the clustering coefficient method, is another critical hub gene. It functions as a transcription factor and is a key regulator of gene expression, particularly in response to viral infection. The activation of TFIIE under stress conditions may enhance the expression of immune-related genes, providing further insight into the plant's adaptive immune response [16]. In the same top-ranking group, FAR1-RELATED SEQUENCE 5 (EL10AC3g06581), another gene ranked first by the clustering coefficient, has been linked to plant development and stress responses. The downregulation of FRS5 upon BCTV infection suggests that viral pathogens may interfere with host defense mechanisms, hijacking cellular resources to facilitate replication [17] (Table 2). Furthermore, EL10AC7g17778 (Probable LRR receptor-like serine/threonine-protein kinase), ranked first by the clustering coefficient, plays a crucial role in plant immune signaling. LRR receptor-like kinases (LRR-RLKs) are involved in recognizing pathogen-associated molecular patterns (PAMPs) and activating downstream immune responses. Studies in other plant species have demonstrated the importance of these kinases in mediating resistance to pathogens, and the identification of this gene in sugar beet highlights its potential role in BCTV defense. LRR-RLKs are known to interact with various immune regulators, including NLR proteins, to coordinate defense responses [18].

**Table 2:** Ranking of hub genes identified in *Beta vulgaris* infected with Beet Curly Top Virus by using CytoHubba.

Rank	Gene ID	Ranking method	Gene description
5	EL10AC7g15913	EPC	Putative malonyl-CoA: Acyl carrier protein transacylase
4	EL10AC6g14637	Bottleneck	Heat shock 70 kDa protein 6, chloroplastic
5	EL10AC7g17862	Bottleneck	Small ubiquitin-related modifier 2
3,4	EL10AC5g12641	Bottleneck, Radiality	Long-chain acyl-CoA synthetase 9
1	EL10AC5g12946	Clustering coefficient	Expansin-like A3
1	EL10AC4g09232	Clustering coefficient	Transcription factor TFIIE, alpha subunit
1	EL10AC3g06581	Clustering coefficient	Protein FAR1-RELATED sequence 5
1	EL10AC7g17778	Clustering coefficient	Probable LRR receptor-like serine threonine-protein kinase at 1g56130

Among the functions of hub genes, long-chain acyl-CoA synthetase 9 (LACS9, EL10AC5g12641), ranked third by the Bottleneck algorithm and fourth by Radiality, was found to be crucial in lipid metabolism. LACS9 facilitates long-chain fatty acid activation and integrates these fatty acids into lipid biosynthesis, maintaining membrane stability under stress conditions. The importance of LACS9 in plant-virus interactions is particularly evident as viruses often exploit host lipid metabolism for replication [19]. Its role in plant defense is well-documented in species like Populus, where lipid remodeling enhances resistance by maintaining membrane integrity [20]. Another essential gene, Heat shock 70 kDa protein 6 (HSP70-6, EL10AC6g14637), ranked fourth by the Bottleneck algorithm, acts as a molecular chaperone, assisting in protein folding and stabilization during stress. Its upregulation during viral infection is critical for maintaining proteostasis and stabilizing client proteins involved in immunity [21,22]. According to the reports, HSP70-6 plays a role in stress adaptation and antiviral defense [23]. This suggests that increased expression of HSP70-6 during BCTV infection may protect sugar beets from proteotoxic stress.

The Small ubiquitin-related modifier 2 (SUMO2, EL10AC7g17862), ranked fifth by the Bottleneck algorithm, highlights the importance of the ubiquitin-proteasome system in antiviral defense. SUMOylation plays a vital role in regulating immune responses, and viral pathogens have evolved to manipulate this system to evade host immunity [24]. For example, Rice stripe virus infection has been shown to alter host ubiquitination patterns, affecting immune components [25]. These findings suggest BCTV may utilize similar strategies to suppress host defense or enhance replication. Among the hub genes identified, EL10AC7g15913 (Putative malonyl-CoA: Acyl carrier protein transacylase), ranked fifth by EPC, is a key player in the synthesis of fatty acids, particularly malonyl-CoA derivatives, which are involved in various metabolic pathways, including the biosynthesis of flavonoids and other secondary metabolites [26,27]. The role of acyl carrier proteins (ACPs) in plant metabolism, particularly in synthesizing complex lipids, has been well documented, but further functional studies are required to elucidate their involvement in viral defense mechanisms [28].

### 3.2 Gene ontology of subnetwork genes that interact with hub genes

**Fig. 3:**
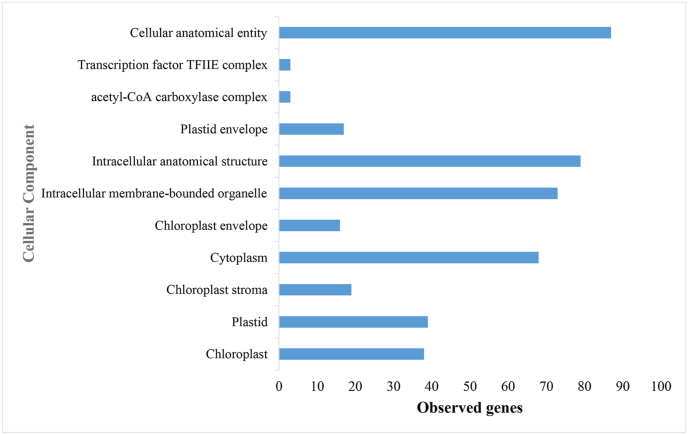
Gene Ontology enrichment analysis (cellular component) of the determined subnetwork genes in *Beta vulgaris* infected by Beet Curly Top Virus using the STRING database.

The integration of GO analysis provides a systematic framework to elucidate the spatial organization of plant responses against viral pathogens. The study of CC in the context of GO highlights the dynamic interplay between various cellular compartments and their roles in plant immunity, particularly under viral stress conditions. In this regard, Fig. 3 presents the CC analysis, emphasizing the significant enrichment of genes associated with the chloroplast. Traditionally recognized for its role in photosynthesis, the chloroplast has recently been identified as a crucial regulator of immune signaling and defense responses in plants, underscoring its dual function and importance in plant immunity [29]. However, viruses such as BCTV exploit this vulnerability by biochemical chloroplast function, thereby compromising both host metabolism and its defense mechanisms [7]. Similar patterns of viral interference have been observed in species such as *Capsicum annuum*, where viral manipulation of chloroplast-mediated responses has been reported [30,31].

These perturbations extend beyond the chloroplast itself, as GO analysis indicates the involvement of other cellular compartments, including cytoplasmic and plastid-related components, in the plant's response to viral infection, suggesting that viral infection affects multiple cellular regions. Notably, the cytoplasm, a hub for various biochemical reactions, is significantly impacted by viral pathogens [32]. Viruses like BCTV establish replication sites within the cytoplasm, hijacking host metabolic pathways to meet their energetic needs. For instance, glycolysis and pyruvate metabolism undergo substantial modifications, ensuring a continuous supply of energy and biosynthetic precursors that support viral replication. While these metabolic alterations benefit the virus, they simultaneously impair the plant's ability to withstand environmental stressors, thus exacerbating the impact of the infection [4,33]. Furthermore, viral proteins interact with intracellular membranes, including those of the endoplasmic reticulum and mitochondria, which facilitates the formation of replication complexes and alters cellular trafficking pathways [34]. This disruption allows the virus to evade host defense mechanisms. Collectively, these findings highlight the extensive reprogramming of the cytoplasmic environment by BCTV, which not only supports its proliferation but also undermines plant immunity (Fig. 3).

**Fig. 4:**
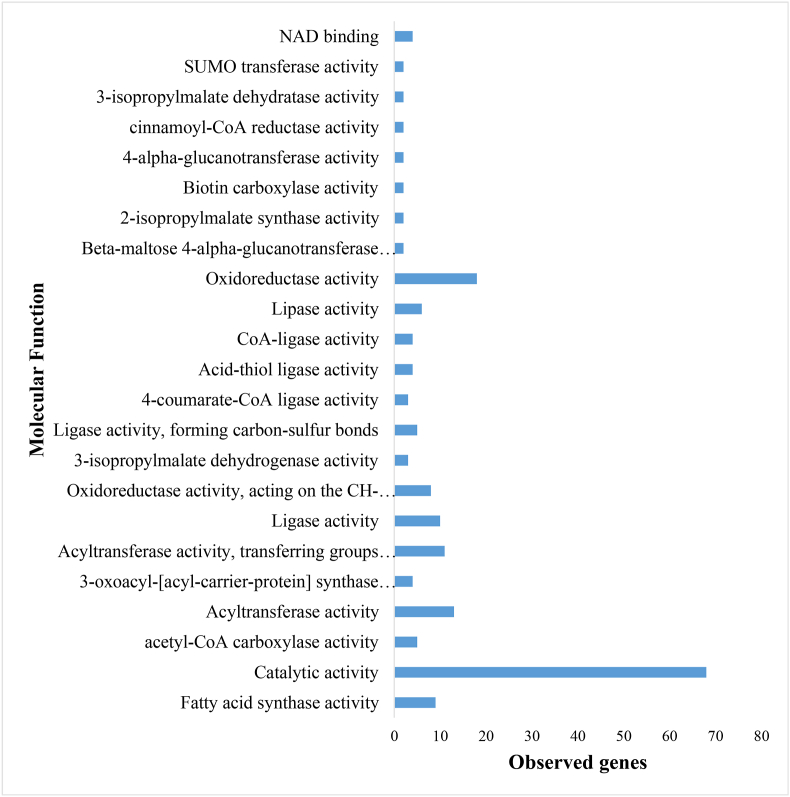
Gene Ontology enrichment analysis of Molecular Function from the determined Subnetwork genes in *Beta vulgaris* infected by Beet Curly Top Virus using the STRING database.

GO MF analysis offers deeper insights into the metabolic shifts induced by viral infections. Fig. 4 illustrates the significant alterations in enzymatic activity, particularly within oxidoreductase and acyltransferase functions, both of which are integral to regulating oxidative stress and lipid metabolism. The observed enrichment of catalytic activity in infected plants emphasizes the essential role of enzymatic defense responses in plant immunity. Viral infections often lead to an accumulation of reactive oxygen species (ROS), which can have dual effects: enhancing antiviral signaling or promoting viral pathogenesis through oxidative damage [35] (Fig. 4).

Lipid metabolism is a primary target of viral interference. BCTV, like many other plant viruses, manipulates acyltransferases to alter the lipid profiles of key organelles, including the endoplasmic reticulum and chloroplasts [36]. These lipid alterations facilitate the formation of viral replication complexes, supporting the hypothesis that lipid metabolism is closely intertwined with both host defense mechanisms and viral propagation. Similar metabolic shifts have been observed in *B. vulgaris*, further highlighting the importance of lipid reprogramming in plant-virus interactions. Moreover, transcriptomic analyses of TYLCV-infected plants revealed the differential expression of over 1000 genes linked to metabolic and catalytic processes, reflecting how viruses manipulate host metabolism to their advantage [5].

**Fig. 5:**
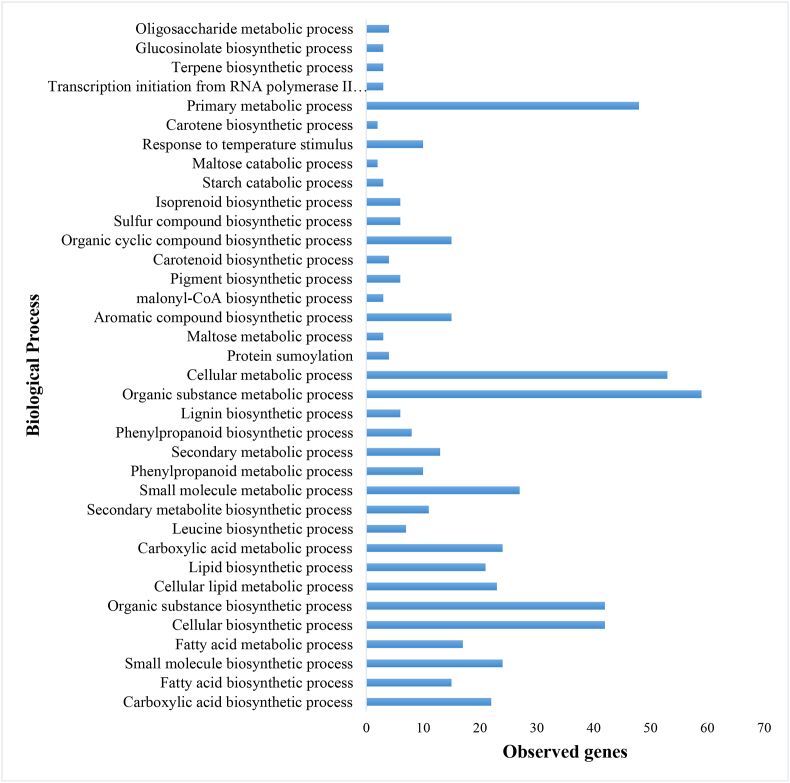
Gene Ontology enrichment analysis of biological processes from the determined subnetwork genes in *Beta vulgaris* infected by Beet Curly Top Virus using the STRING database.

Fig. 5 illustrates the broader BP influenced by BCTV, particularly the enrichment of primary metabolic pathways and biosynthetic processes such as transcription initiation from RNA polymerase II promoter, protein sumoylation, and cellular lipid metabolic process. Viral infections extensively reshape host metabolism, as evidenced by GO analysis, which highlights significant changes in biosynthetic and organic substance metabolism. Similar metabolic reprogramming has been documented in plants infected with *Potato virus Y* (PVY), where transcriptomic studies revealed that host metabolism is systematically altered to accommodate viral genome replication and protein synthesis [37].

**Fig. 6:**
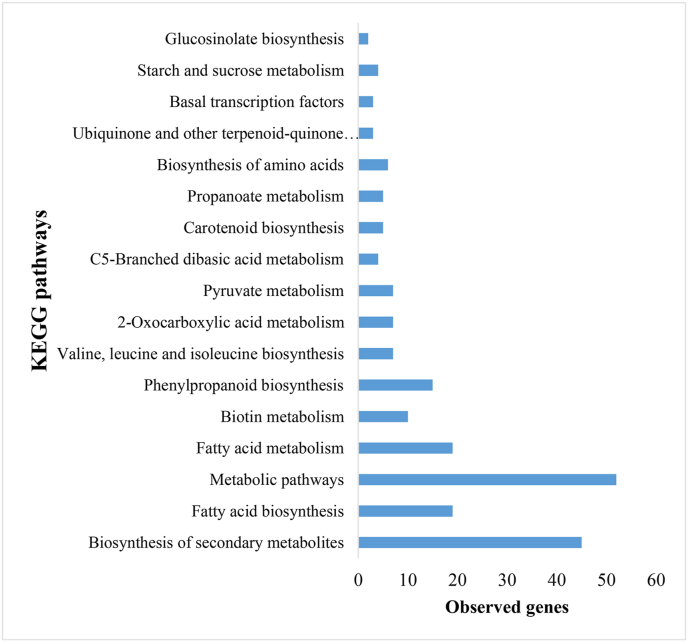
The biological pathways identified according to the Kyoto Encyclopedia of Genes and Genomes (KEGG) on subnetwork genes in *Beta vulgaris* infected by Beet Curly Top Virus using the STRING database.

KEGG pathway analysis revealed significant enrichment of secondary metabolite biosynthesis pathways in response to BCTV infection, with these compounds being strongly associated with antiviral defense through restriction of viral replication and systemic movement. Concurrently, perturbations in lipid biosynthesis pathways were observed, including altered fatty acid metabolism linked to defense signaling. Fig. 6 highlights the role of secondary metabolite biosynthesis in plant defense.

Large-scale metabolic shifts, similar to those seen in BCTV infections, have been reported in *N. benthamiana* infected with Tobacco curly shoot virus (TbCSV), where disruptions in energy production and biosynthetic pathways were evident (Li et al., 2018). A particularly striking response in BCTV-infected sugar beet is the enrichment of secondary metabolite biosynthesis. These bioactive compounds, including phenolics, alkaloids, and terpenoids, play a crucial role in plant defense by restricting viral replication and movement [38]. Further supporting this, metabolomic analyses of *Cucumis sativus* infected with Cucurbit chlorotic yellows virus (CCYV) demonstrated significant enrichment in flavonoid and phenolic pathways, reinforcing their essential role in plant-virus interactions [39]. Although fatty acid biosynthesis showed a lower level of enrichment compared to other pathways, it remains a critical component of plant defense. Fatty acids serve as precursors for signaling molecules such as jasmonic acid, which has a well-documented role in mediating resistance to plant pathogen infections [40]. Disruptions in lipid biosynthesis have been linked to altered viral pathogenesis, as observed in *Oryza sativa* infected with Rice black-streaked dwarf virus (RBSDV), where significant changes in phospholipid and sphingolipid metabolism were recorded [25]. Additionally, the phenylpropanoid biosynthesis pathway, while moderately enriched, plays a key role in lignin, flavonoid, and other phenolic compound production, essential components of structural reinforcement and defense signaling [41]. Similar protective responses have been documented in *Zea mays* infected with Sugarcane mosaic virus (SCMV), where lignin accumulation strengthened resistance to viral infection [42]. These findings underscore the critical role of secondary metabolites and lipid biosynthesis pathways in the plant's defense mechanism against viral infections, highlighting potential targets for improving resistance strategies in crop species.

### 3.3 Promoter motif analysis of hub genes

Understanding the transcriptional regulation of plants in response to viral infections is essential for developing effective strategies to enhance resistance. In particular, studying upstream regulatory regions (UFRs) of hub genes provides insights into conserved motifs and *cis*-regulatory elements (CREs) that orchestrate stress responses. Through MEME analysis, highly significant motifs were identified, many associated with transcription factor (TF) binding sites linked to metabolic regulation and stress adaptation (Table 3).

**Table 3:** The conserved motifs found in promoters of hub genes by the MEME suite. Analysis using the GOMo tool.

Motif	Logo	Predictions	Top 5 specific predictions
MA0008.3		1	CC endomembrane system
MA0097.1		9	MF chlorophyll binding CC chloroplast thylakoid membrane, light-harvesting complex, and mitochondrion BP photosynthesis
MA0584.1		3	MF transcription factor activity CC endomembrane system BP regulation of transcription
MA0932.1		4	MF transcription factor activity CC endomembrane system BP regulation of transcription
MA0951.1		3	MF transcription factor activity CC endomembrane system BP regulation of transcription
MA1161.1		2	MF transcription factor activity
MA1191.1		3	MF transcription factor activity CC endomembrane system BP regulation of transcription
MA1267.1		6	MF transcription factor activity CC nucleus and plasma membrane BP regulation of transcription, DNA-dependent
MA1268.1		7	MF transcription factor activity CC plasma membrane and nucleus BP regulation of transcription, DNA-dependent MF kinase activity
MA1274.1		5	MF transcription factor activity CC plasma membrane and nucleus BP regulation of transcription
MA1277.1		12	MF transcription factor activity CC nucleus and plasma membrane BP regulation of transcription, DNA-dependent and transmembrane receptor protein tyrosine kinase signaling pathway
MA1278.1		7	MF transcription factor activity CC plasma membrane and nucleus BP regulation of transcription, DNA-dependent and response to water deprivation
MA1279.1		6	MF transcription factor activity CC plasma membrane and nucleus BP regulation of transcription, DNA-dependent
MA1281.1		8	MF transcription factor activity CC plasma membrane and nucleus BP regulation of transcription, DNA-dependent and response to water deprivation
MA1328.1		3	MF transcription factor activity CC plasma membrane and endomembrane system
MA1330.1		3	MF transcription factor activity CC endomembrane system BP regulation of transcription
MA1379.1		5	MF transcription factor activity and pseudouridine synthase activity CC endomembrane system BP auxin-mediated signaling pathway
MA1380.1		6	MF transcription factor activity BP response to ethylene stimulus, response to abscisic acid stimulus, response to salt stress and response to water deprivation
MA1672.1		1	CC chloroplast
MA1823.1		10	MF transcription factor activity CC plasma membrane and nucleus BP response to water deprivation and regulation of transcription, DNA-dependent

Among these motifs, MA1277.1 showed the highest prediction and was present in 12 hub genes. It has a predominant association with transcription factor activity as well as its enrichment in the nuclear and plasma membrane compartments. Moreover, this motif was linked to the transmembrane receptor protein tyrosine kinase signaling pathway, which plays a central role in stress perception and immune responses [43]. These findings align with previous studies on plant stress responses, where protein kinases have been implicated in modulating abiotic stress resilience, such as responses to drought, salinity, and cold stress [44]. Similarly, in geminivirus-infected plants, protein kinases were observed to regulate immune pathways, enhancing resistance to viral pathogens [45]. Another notable motif, MA1823.1, identified in 10 hub genes, suggests strong associations with transcription factor activity and responses to water deprivation. Its enrichment in plasma membrane-associated transcription factors reinforces its role in early pathogen recognition and signal transduction, a mechanism previously observed in *A. thaliana*. Specifically, the transcription factor NAC091, located in the plasma membrane, has been shown to regulate the unfolded protein response by transmitting ER-to-nucleus signals under stress conditions [46].

Additional motifs, including MA1267.1, MA1268.1, and MA1274.1, were frequently identified across multiple genes, indicating their potential role as core regulators of transcriptional responses. Their enrichment in nuclear and plasma membrane regions suggests a coordinated mechanism in gene expression regulation during viral infection. Of particular interest is the presence of auxin-mediated signaling motifs (e.g., MA1379.1), which highlights the involvement of hormonal pathways in plant-virus interactions. Previous research has suggested that viruses exploit auxin signaling to promote their replication and systemic movement, as evidenced by viral proteins that disrupt auxin-regulated transcription to facilitate infection [47]. Furthermore, motifs such as MA1380.1, MA1278.1, and MA1281.1 were found to be enriched in genes associated with ethylene and abscisic acid responses, as well as tolerance to salt stress and water deprivation. These findings underscore the relevance of these motifs in plant stress adaptation mechanisms. Ethylene and abscisic acid pathways are widely recognized for their roles in abiotic stress tolerance and pathogen defense responses [48]. Moreover, the identification of chloroplast-associated motifs (e.g., MA0097.1, MA1672.1) suggests a possible regulatory function in photosynthesis and plant defense responses. This observation aligns with research by SU Huh [49] (2024), which demonstrated that chloroplastic ferredoxin, in coordination with Dnaj proteins, plays a vital role in enhancing resistance to viral infections such as cucumber mosaic virus (CMV) by maintaining chloroplast functionality. In the context of *B. vulgaris* defense against BCTV, these motifs likely contribute to sustaining photosynthetic efficiency while activating chloroplast-associated immune responses, reinforcing their significance in plant-virus interactions.

### 3.4 Validation of some of the hub genes using qRT-PCR

In this study, hub genes associated with viral infection in sugar beet were randomly selected and analyzed to understand their role in disease response and their connection to the previous study. qRT-PCR analysis revealed significant upregulation of three key hub genes—EL10Ac7g15913 (Su1/EMB3147), EL10Ac3g06581 (Su2/FRS5), and EL10Ac5g12641 (Su3/LACS9)—in response to viral stress. Our study employed qRT-PCR as a precise and reliable method to validate the expression of selected hub genes during viral infections. This approach mirrors the work of a study [50] that utilized qRT-PCR for the genome-wide identification and validation of reference genes in tomato leaves infected with pathogenic organisms. Also, these findings align with those of Majumdar et al. (2022), who noted a similar expression pattern in response to viral challenges in sugar beet, albeit with a different set of genes. They employed RNA-Seq to uncover genetic responses, highlighting the importance of high-throughput sequencing in identifying potential candidates for viral resistance [10]. Furthermore, the selection of BCTIV for this study was based on its genomic similarities with BCTV, particularly in conserved virulence factors including Rep proteins, which are known silencing suppressors [12].

**Fig. 7:**
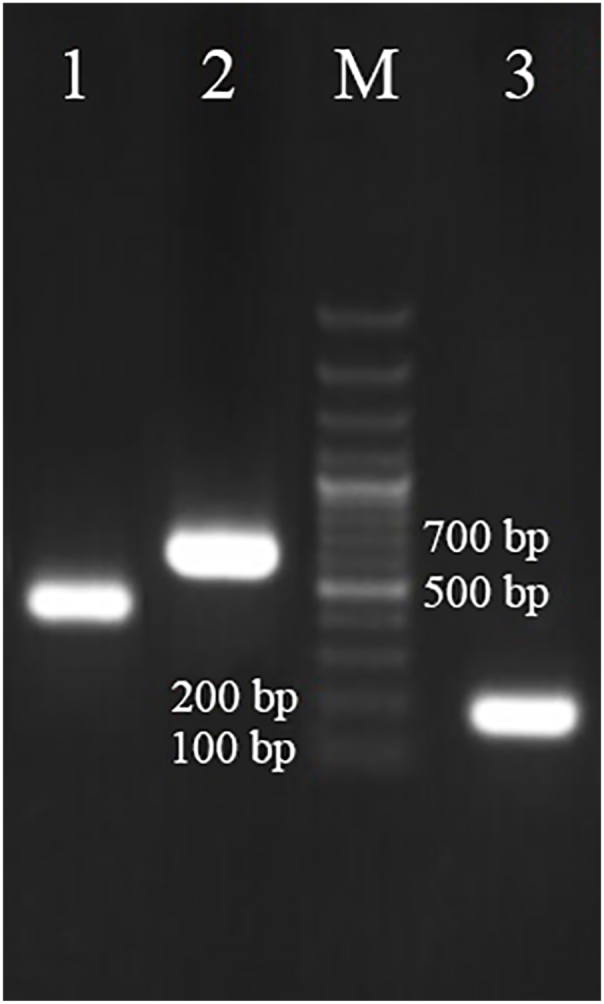
Amplification results of Beet Curly Top Virus and Beet Curly Top Iran Virus genes on a 1 % agarose gel. Lanes 1, 2, and 3 correspond to PCR products amplified using primers specific for Beet Curly Top Virus, Beet Curly Top Iran Virus, and 18S rRNA, respectively. M: 100 bp size marker.

Systemic infection of sugar beet seedlings with BCTV and BCTIV was confirmed via DNA extraction, conventional PCR, and real-time PCR. High-purity DNA (A260/A280: 1.8–2.0) ensured the absence of contaminants, while virus-specific primers yielded distinct amplification bands for BCTV, BCTIV, and 18S rRNA (Fig. 7). The systemic spread was evident by one week post-inoculation, coinciding with severe leaf curling, a hallmark phenotype of these infections. Based on this progression, the one-week time point was selected for downstream gene expression analysis.

**Fig. 8:**
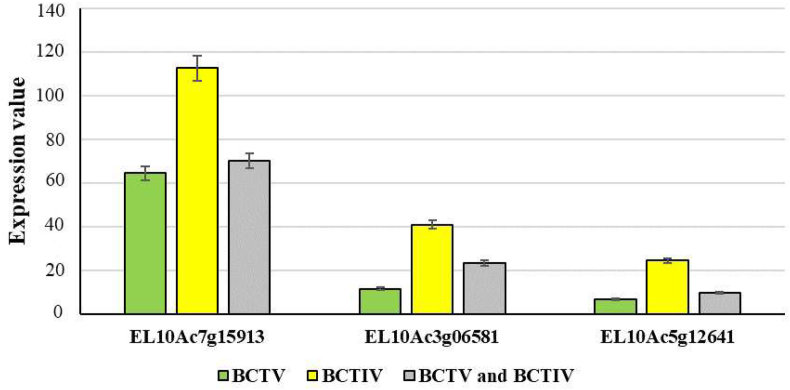
Relative expression diagram of Su1 (EMB3147), Su2 (FRS5) and Su3 (LACS9) genes under the influence of Beet Curly Top Virus and Beet Curly Top Iran Virus infection.

The qRT-PCR analysis revealed significant upregulation of the three key hub genes in response to viral stress. The highest expression levels were observed under BCTIV infection, with a 2.5–3.8-fold increase relative to the control, followed by co-infection (1.9–3.2-fold) and BCTV alone (1.5–2.7-fold) (Fig. 8). These results demonstrated that co-infection led to distinct gene expression profiles, reflecting the complex dynamics of host-pathogen interactions. These findings are consistent with [51], who highlighted the modulatory role of co-infections in influencing disease severity and host responses. They noted that simultaneous infections often alter key regulatory pathways, reshaping gene expression to either exacerbate or mitigate pathogen impacts.

**Fig. 9:**
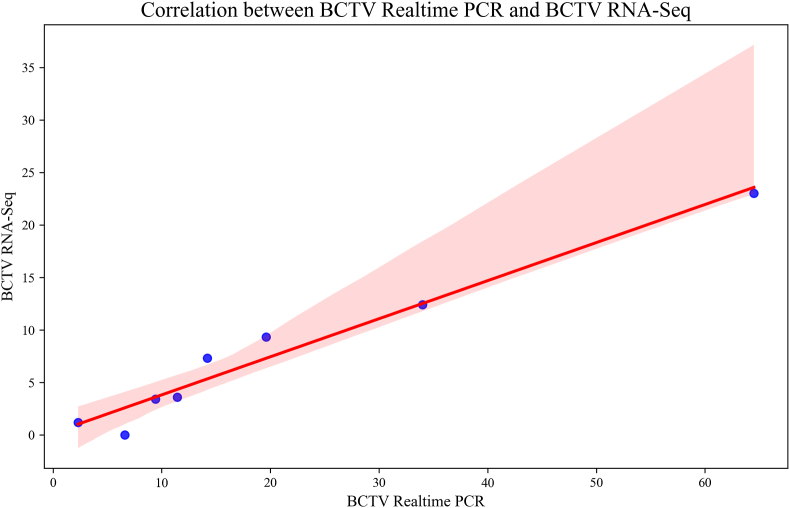
Correlation between qRT-PCR reaction results with RNA-Seq results for Beet Curly Top Virus between hub genes. Correlation coefficient: 0.98.

The bioinformatics predictions were validated by a strong correlation (R^2^ = 0.98) between RNA-seq and real-time PCR data for BCTV (Fig. 9), confirming the reliability of the computational pipeline in identifying key hub genes. The strong correlation between RNA-Seq and qRT-PCR data in our study underscores the reliability of the observed gene expression profiles [52]. Similarly highlighted the effectiveness of integrating bioinformatics approaches with experimental validation to identify critical stress response pathways in plants was highlighted. This integration ensures precision in quantifying gene expression and strengthens the robustness of genetic studies in plant virology.

Given that selected genes showed significant expression in both viral infections, it is plausible that the entire network of predicted hub genes contributes to the host's viral response. These results provide strong evidence that the identified hub genes are not only critical for BCTV response but are also highly relevant in the context of BCTIV infection. This highlights the conserved nature of stress response pathways across different viral strains. Our study emphasizes the conserved nature of stress response pathways across various viral strains. This aligns with [53], who documented the interconnected roles of biotic and abiotic stress responses in shaping plant defense mechanisms. Their research highlighted the activation of shared molecular pathways, which contribute to the resilience of diverse plant species under viral and environmental stress conditions. These findings underscore the potential of collaborative research into host-pathogen interactions to advance disease management strategies across multiple crops. Furthermore, the high correlation observed between bioinformatics and experimental data underscores the effectiveness of integrating computational predictions with laboratory validation, reinforcing the accuracy and robustness of the identified molecular pathways.

## 4 Conclusion

In this study, the molecular and metabolic responses of *B. vulgaris* to BCTV infection were explored using bioinformatics tools such as STRING, GO, KEGG pathway analysis, and promoter motif analysis to identify key hub genes and their associated pathways. The results of this study demonstrate that significant changes in the metabolic pathways of Beta vulgaris were induced by BCTV, particularly in primary metabolism and biosynthesis of organic compounds. Notable alterations in nucleotide metabolism, amino acid synthesis, and secondary metabolite pathways, such as phenolics, alkaloids, and terpenoids, were highlighted by GO and KEGG analyses, which play crucial roles in plant defense against viruses. Furthermore, it was revealed through promoter motif analysis that many of these genes are influenced by hormonal signaling pathways like ethylene and abscisic acid. It is suggested that plant defense responses to viral infections involve a complex interplay of metabolic regulation and hormonal signaling, a mechanism that has been observed across various plant species. Finally, significant upregulation of key hub genes, such as EMB3147 and LACS9, was confirmed through qRT-PCR results, reinforcing the crucial role of these genes in plant viral infection.

## CRediT authorship contribution statement

**Zeinab Porameri:** Writing – original draft, Software, Methodology. **Abozar Ghorbani:** Writing – review & editing, Validation, Supervision, Conceptualization. **Zahra Mirsoleymani:** Supervision. **Marzieh Karimi:** Writing – review & editing. **Mahsa Rostami:** Writing – review & editing, Validation. **Seyed Ali Hemmati:** Writing – review & editing.

## Funding sources

This research did not receive any specific grant from funding agencies in the public, commercial, or not-for-profit sectors.

## Declaration of competing interest

The authors hereby declare that there are no conflicts of interest, financial or otherwise, that could influence the design, execution, or interpretation of this research. No funding sources, commercial entities, or personal relationships have influenced the outcomes or conclusions of this study. The research was conducted independently, with the sole aim of advancing knowledge and developing tools for agricultural diagnostics. All authors have approved this declaration and confirm that the manuscript represents an honest and unbiased account of the research conducted.

## Data Availability

No data was used for the research described in the article.
